# Diagnostic Challenges in the Early Onset of Inflammatory Bowel Disease: A Case Report

**DOI:** 10.22088/IJMCM.BUMS.7.4.251

**Published:** 2019-02-28

**Authors:** Naghi Dara, Sharam Nemati, Sharam Teimourian, Farid Imanzadeh, Amirhossein Hosseini, Saleheh Tajalli, Ali Akbar Sayyari, Ali Najafi, Pejman Rohani, Katayoun Khatami, Elahe Motevaseli, Martin de Boer, Taco W. Kuijpers

**Affiliations:** 1 *Pediatric Gastroenterology, Hepatology and Nutrition Research Center, Research Institute for Children's Health, Shahid Beheshti University of Medical Sciences, Tehran, Iran.*; 2 *Department of Medical Genetics, Tehran University of Medical Sciences, Tehran, Iran.*; 3 *Department of Medical Genetics, Iran University of Medical Sciences, Tehran, Iran.*; 4 *Student Research Committee, Iran University of Medical Sciences, Tehran, Iran.*; 5 *Molecular Biology Research Center, Systems Biology and Poisonings Institute, Baqiyatallah University of Medical Sciences, Tehran, Iran.*; 6 *Department of Molecular Medicine, School of Advanced Technologies in Medicine, Tehran University of Medical Sciences, Tehran, Iran.*; 7 *Sanquin Blood Supply Organization, and Landsteiner Laboratory, Academic Medical Centre, University of Amsterdam, Amsterdam, The Netherlands.*

**Keywords:** Early onset inflammatory bowel disease, genetic, IL-12RB1 deficiency, pediatric

## Abstract

Inflammatory bowel disease (IBD) with very early onset manifestations (younger than six years of age) is an essential pediatric gastrointestinal disease that encompasses a group of diverse and rare genetic defects. It may be associated with chronicity, premalignant nature, and high morbidity and mortality during childhood. Because of overlapping phenotypes, the definitive diagnosis based on conventional strategies is frequently a challenge. However, many patients with different molecular pathologies are treated with the same therapeutic strategy. In this context, it is essential to define a more reliable method to provide an opportunity for a rapid and accurate diagnosis. Here we report a novel homozygous exonic variant in a patient with an IBD-like lesion in the colon during the infancy period. A 7 months old boy who was born of a consanguineous marriage developed gastrointestinal disorders early in life. After complete diagnostic workups, this case underwent conventional therapy of IBD for five months; but clinical remission was not achieved. We identified a novel homozygous mutation (c.684C>T p(=)) in exon 7 of *IL-12RB1* gene that *in silico* studies indicated its significance in the splicing process. At the 14^th^ month of age, this case died. Our finding reveals the importance of genetic screening as an early diagnostic tool in the identification of the underlying causes of IBD with very early onset manifestations, particularly infantile (< 2 years of age) IBD. This strategy makes an opportunity in prompt diagnosis and targeted therapy.

Many inflammatory disorders involving gastrointestinal (GI) tract manifest themselves early in life such that they are indistinguishable from each other only by the classical approaches (1). It is well documented that in patients with primary immunodeficiency, GI inflammation is the first and only manifestation for a long time (2). Indeed, the gut inflammation is a common feature in chronic granulomatous disease (CGD) (3), X-linked agammaglobulinemia, severe combined immunodeficiency (SCID), DiGeorge syndrome, X-linked lymphoproliferative disease (XLP), Wiskott-Aldrich syndrome (4), X-linked inhibitor of apoptosis (XIAP) (5), interleukin 10 receptor (IL-10R) deficient patients (6) and so many others. These subtypes make a small fraction of pediatric inflammatory bowel diseases (PIBD), but they have particular phenotypic and genetic characteristics accompanied by a severe disease course and inadequate response to conventional therapy. In this study, we describe a boy manifesting GI disease during infancy, who was hospitalized for intractable diarrhea. Interestingly, the preliminary diagnostic workups were in favor of IBD, but genetic screening showed that he had a novel exonic variant in *IL-12RB1*.

## Case presentation

**Fig. 1:**
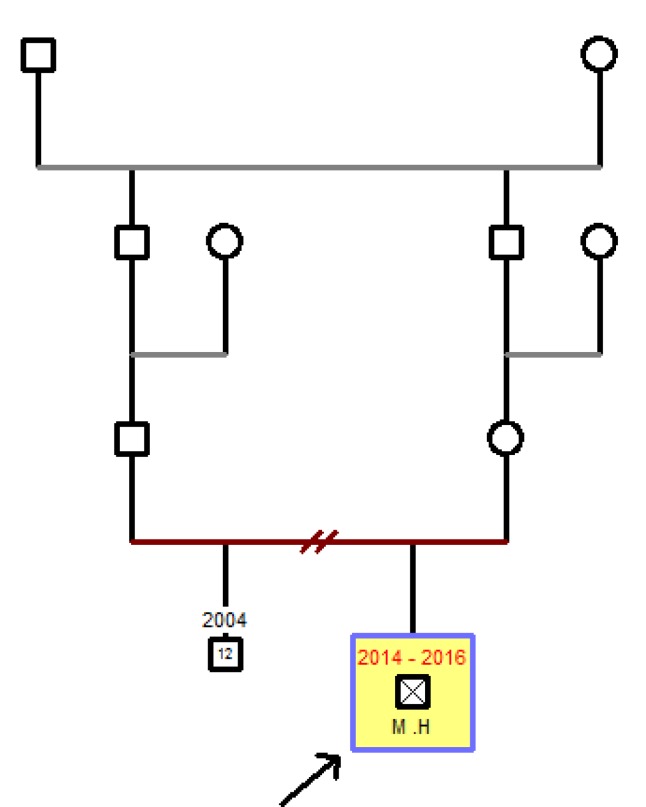
Family pedigree with consanguineous healthy parents

**Fig. 2:**
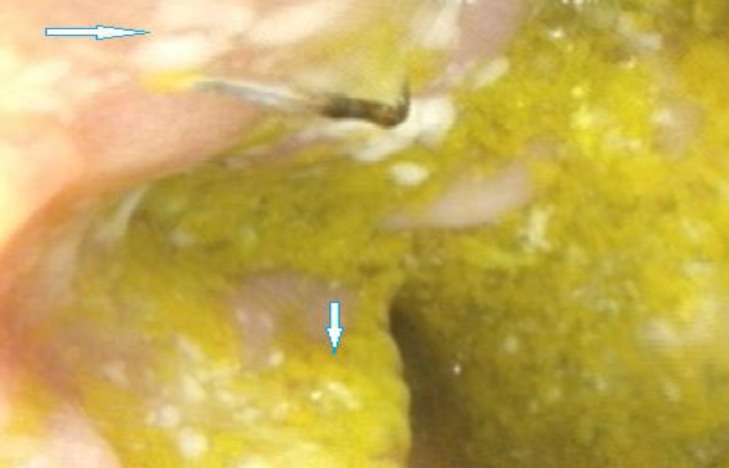
**Endoscopic features of the patient carrying variants in IL-12** **Receptor gene.** Upper arrow shows ulceration lesion, whitish exudates, and distorted vascular pattern in colonic mucosa; lower arrow shows fecal material

**Fig. 3:**
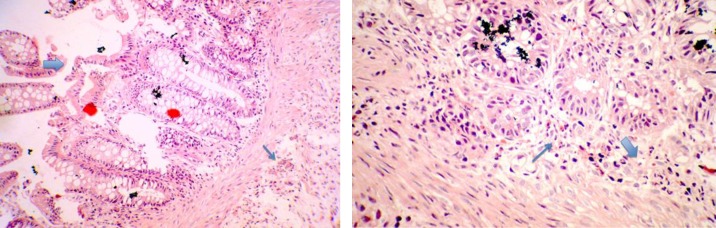
**Histopathologic results** **of the patient carrying variants in IL-12 receptor gene** **. **A: undulated surface epithelium with mildly disorganized glands (thick arrow), increased eosinophils (thin arrow) stained with hematoxylin and eosin (x200); B: neutrophilic infiltration with increased eosinophils stained with hematoxylin and eosin (x400)

We reported a seven month of age boy born to consanguineous healthy parents (Figure 1). He was hospitalized at the age of 20 days for neonatal jaundice. In the second month of life, he presented watery diarrhea, which after two weeks changed quality to bloody mucoid diarrhea, and because of its intractable nature, he was transferred to the pediatric gastroenterology unit at 4.5 months of age. A complete diagnostic approach and workup was performed; including physical examinations, laboratory assays consisting of immunological, hematological, microbiological and radiologic tests. Upper and lower gastrointestinal video endoscopy with biopsy were performed. The hematologic findings revealed non- hemolytic anemia that was attributed to bleeding from the GI tract. Initial workup for immune system assessment including quantification of immuno-globulins level (IgG, IgA, IgM, and IgE), evaluation of adaptive arms of immune system, and lymphocyte panel tests (T cell, B cell, CD4^+^T and CD8^+^T cells), all of them were normal except the insufficient level of serum IgG. All virology markers for TORCH, cytomegalo-virus and Epstein- Barr virus were negative. Colonoscopy showed proctocolitis (Figure 2), and histopathologic results revealed mild chronic crypt destructive inflammation with mild activity and severe eosinophilic infiltration (Figure 3).

**Fig. 4:**
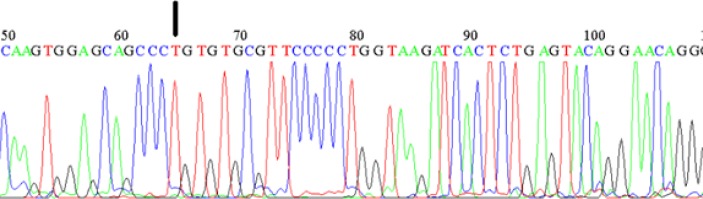
**Sanger sequencing analysis of** ***IL-12RB1*** **. **The presence of c.684 C>T substitution at the end of exon three confirms the preliminary screening of this gene by next generation sequencing

Therefore, this case with the early diagnosis of allergic colitis underwent a therapeutic measure including amino acid- based formula, complemen-tary nutrients, and prednisolone. After five months, our case did not respond to medical therapy, and developed failure to thrive (FTT). At this stage, very early onset IBD was considered as differential diagnosis.

**Identification a novel homozygous variant in** ***IL-12RB1***

Five to 50 % of patients with primary immune deficiency manifest gut inflammation as the first feature for several years. Thus, we screened the genome of the patient for detection of potential mutations in genes having a known and essential role in IBD pathogenesis; including *IL-10, IL-12, IL-23, IL-27, *and *INFγ *(7-9). Then, we used next generation sequencing (NGS) technique to sequence genes of interest belonging to Thermo Fisher immunodeficiency panel. These include coding sequences (CDS) of *IL-12 *related genes (*IL-12A, IL-12B, IL-12RB1, IL-12RB2*), *IL-10* related genes (*IL-10RA, IL10RB, IL-10*), *INFG* related genes (*INFGR1, INFGR2, IFNG*), *IL-23P19, *IL*-23R, IL-27A (P28), WSX1 (IL-27RA)*. The NGS revealed novel homozygous (c.684C>T p.(=)) variant lying in the exonic region near the end of the exon 7 of *IL-12RB1*. This result was confirmed by the Sanger manual sequencing method (Figure 4). Because at the time of receiving the result of NGS the case died at the 14 months of age, it was not possible to perform the functional study, and we decided therefore to use some tools to predict the functional impact of the variant on the gene function. *Phylogenetic P*-*values*, which is a tool for measuring evolutionary conservation of sites by scoring them from -14 to +3, revealed that our target location was conserved across evolution. The decisive score predicts the conservation and vice versa. Our target site got number 2.05 score. Based on the location of the variant; we supposed that this region could be crucial in the splicing process. During this process, in which some conserved nucleotides have an irreplaceable role, non-essential parts of primary transcript are snipped out.

**Fig. 5:**
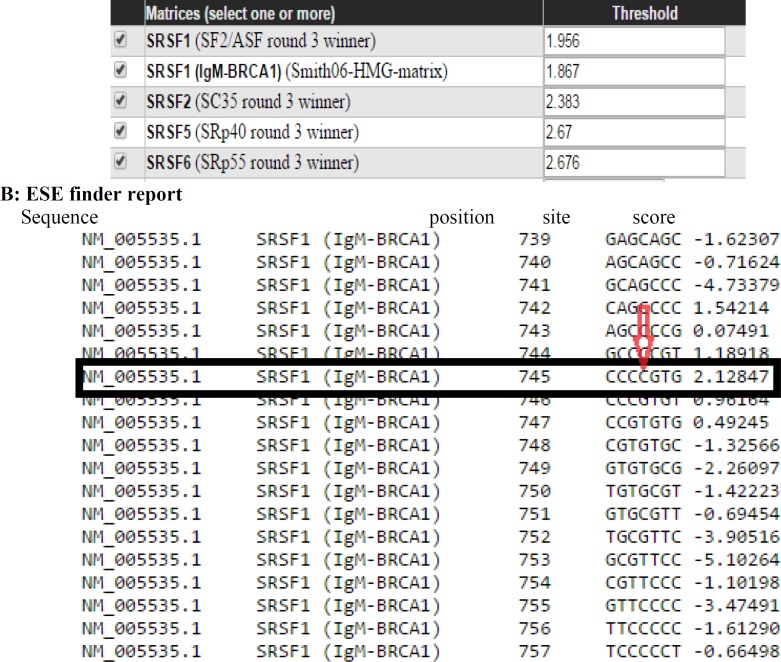
**The accepted thresholds for specific serine/arginine (SR) proteins. **A: analysis of query sequences thought to be significant in the splicing process. The data included in this table show different types of SR family proteins and their threshold. The thresholds are values above which a given sequence is considered to be significant (high-score motif). The default threshold values are set as the median of the highest score for each sequence; B: ESE finder provides a prediction of the functional effect of different sequences having seven nucleotides long. As shown, the c.684C>T substitution lies in the region having a score that exceeds the threshold identified in the table for SRSF1 (IgM-BRCA1). It implies that this sequence may be a binding site for proteins that are crucial in splicing, and therefore it can be postulated that any variants interrupting this process could have a deleterious effect on gene function

**Fig. 6:**
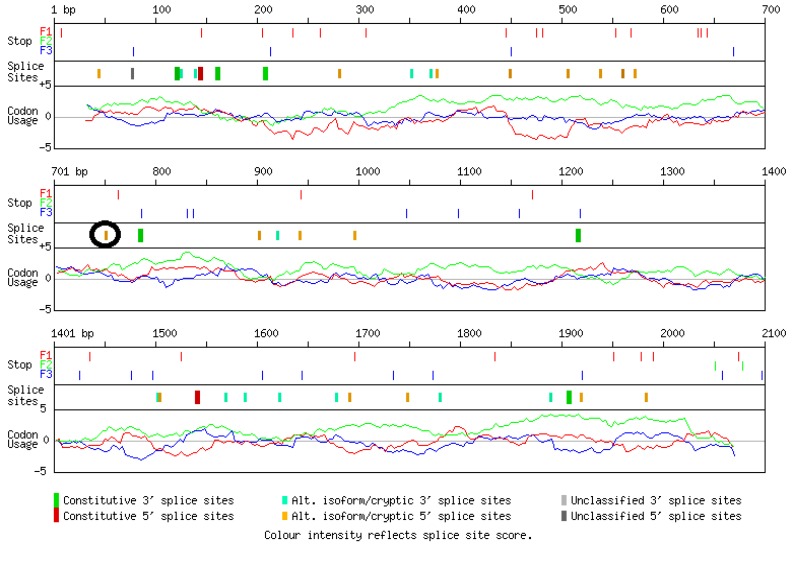
**Alternative splicing site prediction (ASSP) as a tool to analyze either donor or acceptor splice sites strength.** If the sequence of splice site surpasses the cutoff value, it is classified as alternative isoform/cryptic or constitutive. c.684C>T was found in a sequence that is predicted to be an alternative cryptic splice site which means it could alter splicing such that it disrupts the gene function. The cutoff value for acceptor site is 2.2, and for donor site is 4.5. F1, F2, F3, show all possible reading frame. As it is characterized, the region around nucleotide 750 is predicted as alternative cryptic 5’ splice site

Splicing is known to be controlled through splice enhancer sequence and splice silencer sequence located in either exons or introns. Exonic splice enhancer (ESE) has about 6-8 nucleotides long known to be the binding site of regulatory proteins. Mutations in these regions have distinct consequences including intron retention or exon skipping that can both disrupt the gene function. Moreover, some sequences have cryptic splice sites that are similar to authentic ones but not generally used in splicing. If a mutation occurs in these places, it might alter a cryptic site such that it would be used in normal splicing, resulting in aberrant products.

To identify the functionality of the region harboring the novel mutation, we used some bioinformatics tools such as F-SNP for finding ESE, and alternative splicing site prediction (ASSP) for cryptic splice sites detection. The sequence was submitted in FASTA format, and then F-SNP reported the best hits as numerical codes. Those values that exceed the thresholds were considered significant (Figure 5 and 6). As expected, these analyses revealed that the corresponding variant was located in a central zone, that statistically might play a significant role in the splicing process. However, this needs to be confirmed by a functional study.

## Discussion

The gastrointestinal disorders with early onset manifestation represent a significant challenge in diagnosis, and subsequently appropriate treatment. In this situation, understanding the underlying molecular pathology may open a promising field for individualized and effective treatment including hematopoietic stem cell transplantation. Because these types of disorders have overlapping phenotypes, the accurate diagnosis based on the classical approach is far from access. As such, many diseases that have different pathogenesis show identical phenotype, making the molecular study prone to selection bias. An inadequate response of the present case to conventional therapy persuaded us to use genetic testing. It was surprising that NGS revealed a homozygous variant in *IL-12RB1* gene. This gene encodes for a type 1 transmembrane protein, and with its counterpart (IL-10RB2) they make high- affinity binding sites for IL-12. Much evidence revealed the crucial role of the IL-12 signaling pathway (IL-12/IL-12R) in the pathogenesis of Crohn's disease where *IL-12 *and *IL-12RB2* are upregulated (10, 11).

Additionally, overexpression of *IL-12RB2* was shown to be associated with Th1- mediated inflammatory disease. IL-12 has a pivotal role in differentiation of naïve T cells to INF-γ/TNF producing Th1 cells by which intestine inflammation ensues (12).

Conspiciously, we identified a homozygous mutation in the conserved region of *IL-12RB1*, and this mutation was predicted to have an adverse impact on gene function. *IL-12RB1* deficiency is a well-known rare autosomal recessive disease accompanied by susceptibility to disseminated infection caused by atypical and poorly pathogenic mycobacterium and salmonella (13, 14). Under normal circumstances, activation of IL-12 signaling pathway via signal transducer and activator of transcription (STAT) 4 culminates in transcription of some target genes such as *INF-γ* that has an essential role in the innate and adaptive immunity against a wide range of microorganisms (15). It is important to note that all individuals carrying a mutation in the *IL-12RB1 *are asymptomatic. In reality, some individuals do not have an adverse response to Bacillus Calmette–Guérin (BCG) vaccination (16) and manifest disease late in life (17). This could be attributed to the redundancy of IL-12 with the weekly virulence activity for protection against pathogen (13). Because of the low clinical penetrance of the underlying mutations, the disease can occur across a broad spectrum of time (from 1 week to 31.7 years) (14). It has been suggested that additional immune deficient predisposing factors or triggers need to be present in order to develop disease (18). Our case had no adverse response to BCG vaccination, and as mentioned, he was admitted to the gastroenterology department for profound diarrhea lasting for several months. It could be postulated that the disability in eradication of intracellular microorganisms may result in the dysregulated gut mucosal immune response to constitute normal microflora although the exact mechanism involved have yet to be established by further research in the future.

Diagnostic workup revealed the inflammation across the gut, and then the case was treated to control diarrhea and long-term mucosal healing achievement. If it it was known that this patient was IL-12RB1 deficient, a different therapeutic approach would have been used. Our work highlights that due to overlapping manifestations of IBD –like intestinal diseases with very early onset, it is necessary to revise the classical diagnostic approach by modifying the starting point from functional studies to genomic screening.

## Conflict of interest

Authors declare no conflict of interest.
